# The Effect of Optimized Substrate Orientation on Layer Step in Laser Metal Deposition of Single-Crystal Nickel-Base Superalloys

**DOI:** 10.3390/ma17184607

**Published:** 2024-09-20

**Authors:** Jiachen Guo, Junxiang Zhou, Yong Sun, Bo Feng, Yunwei Zhang, Chang Ding

**Affiliations:** Shaanxi Key Laboratory of Artificially Structured Functional Materials and Devices, Air Force Engineering University, Xi’an 710051, China; flyjunxiang@126.com (J.Z.); fengbo@buaa.edu.cn (B.F.); zhang_yunwei@126.com (Y.Z.); dingchanghit@alu.hit.edu.cn (C.D.)

**Keywords:** single-crystal superalloys, substrate orientation, stray grain susceptibility, laser repair

## Abstract

Laser metal deposition is a promising way to repair the surface defects of single-crystal components in turbo engines. Understanding the mechanisms and improving the efficiency of the repair have been long-standing problems. In this study, the influence of the substrate orientation on the laser metal deposition (LMD) was investigated and its effect on repair layer-step was examined. LMD experiments were conducted on single crystal superalloys with a normal substrate orientation (001)/[100] and with an optimized substrate orientation (101)/[10$\bar{1}$]. It reveals that the laser cladding with the optimized orientation leads to a larger height of the [001] dendrite region than that with the normal orientation. The calculated results of the growth velocity, thermal gradient, and susceptibility to CET in the dendrite-preferred growth direction indicate that, for the (101)/[10$\bar{1}$] orientation, the [001]/[100] boundary is located at relative high position in each layer, which not only decreases the formation ability of stray grain significantly, but also eliminates the appearance of the maximum susceptibility. This makes the necessary dilution position much higher, and thus, a large cladding step can be selected. Our findings could find potential applications in laser repair of single-crystal components.

## 1. Introduction

**Figure 1:**
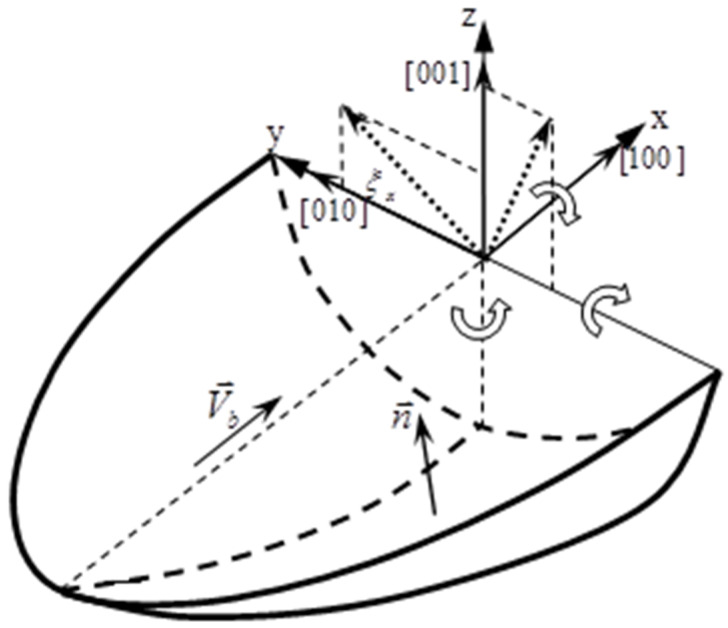
Schematic illustration of the substrate orientation used in this work.

According to previous studies [1,2,3,4,5], during the laser remelting process of the single-crystal (SX) Ni-base superalloy, the formation of equiaxed stray grains can be influenced by the rotations of substrate orientation around different axes. Defined an initial substrate as shown in Figure 1, the rotation of the substrate around [010] crystallographic directions by 45° can suppress the stray grain effectively. This paper will apply this effect to the epitaxial laser metal deposition (E-LMD) process, by which we shall show that the layer step can be increased and it could improve the efficiency of repairing SX components. 

For a successful SX crystal repair, two aspects should be considered [6,7]. One is that the deposited part must maintain the same crystal orientation as that in the substrate [6]. Otherwise, the breakdown of the single-crystal nature will take place, resulting in a failed repair. The main factor leading to the non-single-crystal nature is columnar-to-equiaxed transition (CET), which induces equiaxed stray grain formation; thus, CET should be avoided during the repair process [8,9,10]. Since CET is mainly controlled by the thermal gradient, *G*, and growth velocity, *V*, within dendrite domainsin melt pools or deposited traces, the repair parameters should be adjusted interactively to guarantee the required range of *V* and *G*. The other aspect is the repair efficiency. This is imperative, since the repair is usually conducted by laser scanning in the velocity range of millimeters per second, and it takes a long time. 

To improve the efficiency of repairing process, except for the laser scanning velocities, the layer step which denotes the height of the unmelted part of the previous laser trace should be selected to be as large as possible. Since the increase in the scanning velocity will increase the dendrite growth velocity, which will not benefit to the avoidance of CET, there is normally a limit to the scanning velocity [11,12]. For this reason, we will focus on the changing of the layer step in this paper. 

The layer step is usually considered to be varied by changing processing conditions, such as laser power, scanning velocity, and powder feeding rate. A common experience is that the increase in the powder feeding rate can increase the layer step [13]; however, the direct relationships between them are complicated. Here, we take into account the simple case of increasing the layer step by changing the substrate orientation, which can be performed under unique processing conditions.

**Figure 2:**
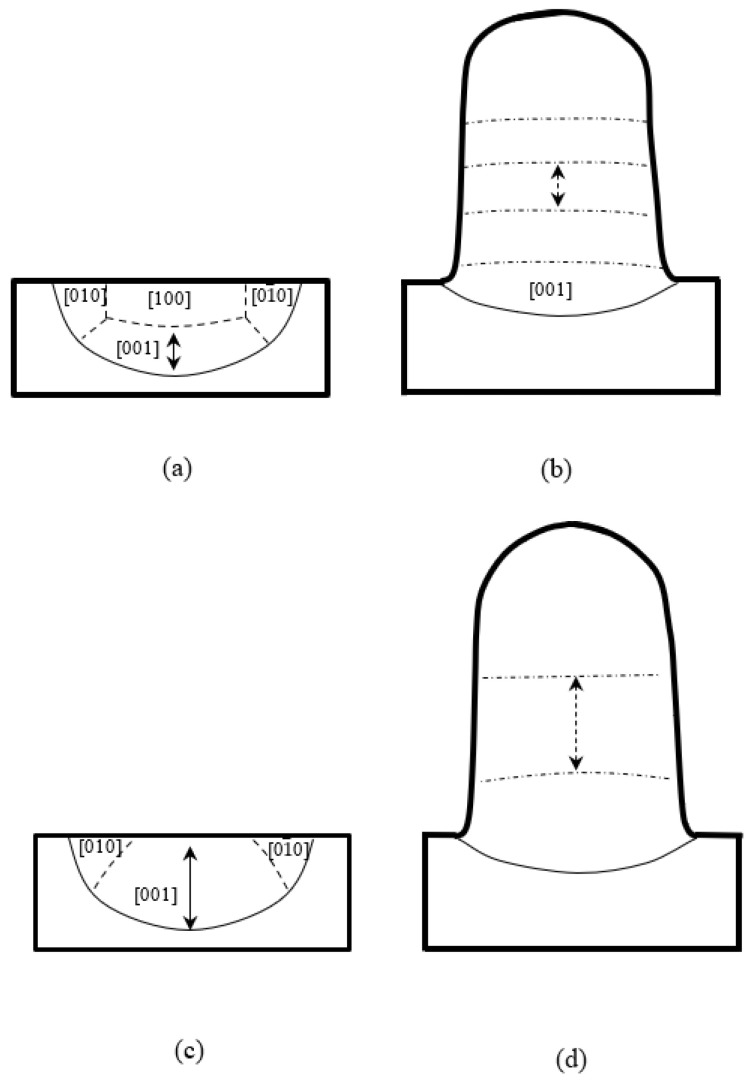
Schematic illustrations of the dendrite domains with different preferred growth directions in transverse section during LR and LMD. Substrate (001)/[100] in (**a**) LR and (**b**) LMD; substrate (101)/[10$\bar{1}$] in (**c**) LR and (**d**) LMD.

The casting of SX-blades is usually along one of <001> axes [14,15]; thus, the laser remelting or laser metal deposition usually proceeds on one {100} surface and the laser beam normally scans along one <001> direction [16,17,18], as illustrated in Figure 1. In this case, the re-solidified melt pool consists of several cell/dendrite domains with different preferred crystalline orientations, as illustrated in Figure 2a. The [001] domain normally keeps the orientation from the substrate and can still have single crystallinity [19,20,21]. The stray grains have been shown to be formed preferentially near the region of the [001]/[100] boundary and in the upper part of the [100] domain. For the LMD, the new layer deposited on the previous one usually requires remelting the previous [001]/[100] boundary; therefore, for a laser repair, the layer step is usually selected to be slightly smaller than the height of the [001] domain for avoiding SGs, as the arrow shows in Figure 2b. It should be noted that during the repairing process, because the main part of the repairing region is on the top of the substrate, most of the heat is conducted downwards by the solid substrate, whereas heat loss at the side boundaries due to air convection is much less efficient. In this case, [010] and $\left[0\bar{1}0\right]$ domains usually do not appear, and only [001] and/or [100] domains exist. For avoiding the formation of stray grains at the [001]/[100] boundary, the height of the [001] region limits the layer step from traveling upwards. One possibility to break such a limit is to increase the size of the [001] domain by changing the substrate orientation. Research shows that the variation in the substrate orientation around the [010] axis can vary the sizes of different domains. When the rotation angle reaches 45°, the [001] domain can extend to the top of the surface and the [001] region occupies the main part of the weld pool, as shown in Figure 2c. If the [001]/[100] boundary does not appear in the LMD process, as shown in Figure 2d, the height of the [001] domain can be expanded and the layer step for LMD can be increased effectively. 

The aim of this work is to clarify this point. The substrate orientation can affect the stray grain formation in laser-remelted single-crystal superalloys. A systematic investigation into the effect of substrate orientation on stray grain susceptibility was performed on the two most conventional crystallographic planes, (001) and (101). We will determine whether the variation in substrate orientation via the [010] axis can improve the efficiency in the laser metal deposition (LMD) process. LMDs will be performed when the substrate orientations are 0°/[010] and 45°/[010] and its effects on the layer height and single crystallinity will be examined. Our results show that stray grain can be depressed most effectively if a laser repair is performed in the (101) plane along the [10$\bar{1}$] direction, which can provide in-depth insight into the mechanism of how to avoid stray grain formation in the future laser repair of single-crystal components.

## 2. Experiments

The second-generation nickel-based superalloy DD6 [22] developed in China was chosen as the substrate material for this study. DD6 superalloy powder of the same material as single-crystal substrate was used as an additive material. Before laser processing, we used an oven to dry the powder. The compositions are listed in Table 1 [23,24,25].

**Figure 3:**
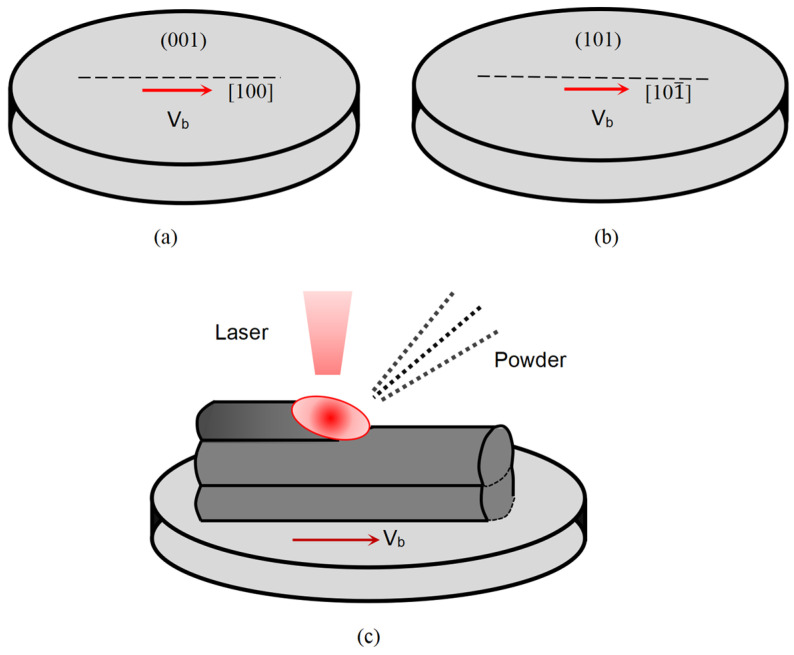
Schematic illustrations of the specimens with substrate orientations of (**a**) (001)/[100], (**b**) (101)/[10$\bar{1}$]; and (**c**) laser metal deposition process.

Specimens in the shape of round plates in the experiment were cut from an SX cylindrical DD6 ingot with a diameter of 15 mm, which was produced by a traditional casting method. With the main axis of the ingot being [001], the specimens were cut after the ingot was rotated around the [010] axes for 0° and 45°, as shown in Figure 3. For these two substrates, marked as A and B, the crystal planes/scanning directions in the LMD process were (001)/[100] and (101)/[10$\bar{1}$], respectively, as given in Figure 3a,b. The (001)/[100] orientation is a normal one which is usually used in LMD, while (101)/[10$\bar{1}$] is an optimized one in this work. The thickness of the samples was 1.5 mm, and the schematic diagram of the LMD process is shown in Figure 3c.

Two sets of experiments were carried out. In the first set, LMD processes were conducted directly on the two substrates with different upgrade steps or z-steps: 100 and 300 μm. z-step here means the levitation step of the laser beam. Please note that due to the different powder feeding rates and other experimental parameters, the layer step is different from the z-step. During the experiment, for the 100 μm z-step, five layers were deposited, while for the 300 μm z-step, three layers were deposited. In the second set of experiments, laser remelting was performed firstly, and then the laser metal deposition process with three-layer deposition was carried out on the remelting trace. We selected a z-step of 300 μm in this case so that the position of the bottom trace of LMD would be higher than the [001]/[100] boundary in the laser remelting trace for substrate A. Under this condition, some stray grains were expected to be produced near the [001]/[100] boundary in the LR trace, and its effect on the following LMD could be checked. 

**Figure 4:**
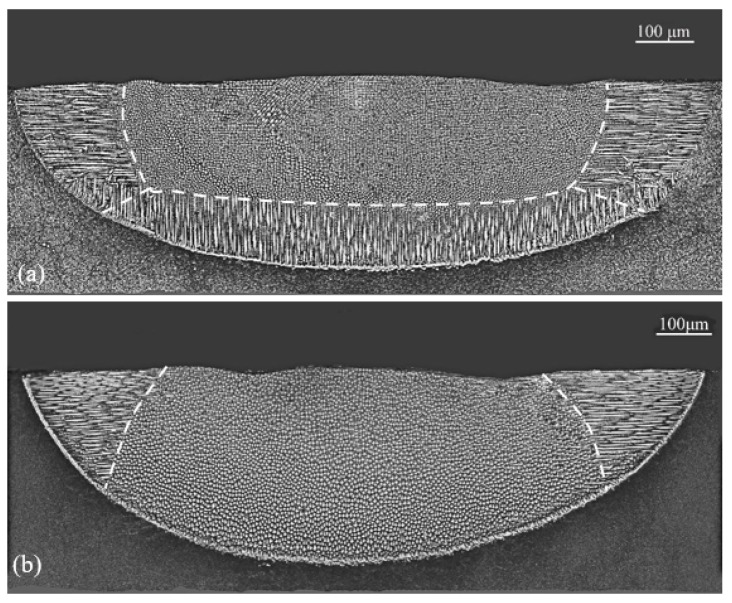
Transverse section microstructures of specimens with substrate orientation of (**a**) (001)/[100] and (**b**) (101)/[10$\bar{1}$] under laser LR conditions of a power of 250 W and a laser scanning velocity of 5 mm/s.

The LR and LMD experiments were performed by using a 1 kW continuous wave Nd: YAG laser. The laser beam was a Gaussian heat source with a radius close to 0.75 mm and a power distribution of 2. To check the effect of the substrate orientation uniquely, we selected a processing condition where the laser power was 250 W and the scanning speed was 5 mm/s, under which the laser remelting was carried out and good single crystallinity was obtained in the [001] region for both substrates, as given in Figure 4. Under this processing condition, the height of the [001] domain was about 150 μm in substrate A (Figure 4a) and about 300 μm in substrate B (Figure 4b). Moreover, some stray grains could be found at the [001]/[100] boundary in the first case. These stray grains will be used to in our second set of experiments. This is the reason why we selected a z-step of 100 or 300 μm in our experiments.

After the experiments, the samples were polished and etched with a solution of diluent royal water (50% HCL, 25% HNO_3,_ and 25% H_2_O in volume ratio). The microstructures of the deposited parts were observed with optical and electron microscopes. The orientation in the LMD part was determined by the electron backscattered diffraction (EBSD) technique.

## 3. Results

### 3.1. z-Step of 100 μm in LMD

**Figure 5:**
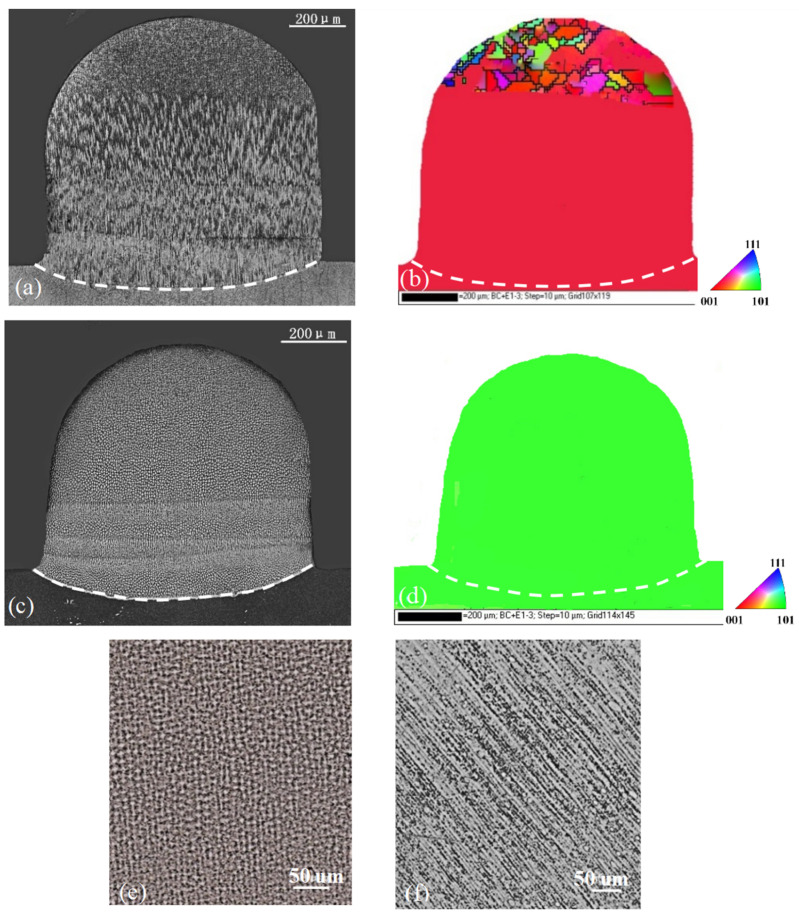
Microstructures and corresponding EBSD maps of substrate A (**a**,**b**) and substrate B (**c**–**f**) when z-step was 100 um for four LMD layers.

Figure 5 gives the optical microstructures and the corresponding EBSD maps of the specimens in the transverse section with substrates A and B for the z-step of 100 μm, in which four layers were deposited with the final dimensions of 0.8 mm height and 0.8 mm width. 

For substrate A, it can be seen that from the diluted lower boundary in Figure 5a, fine dendrites grew along the original crystallographic orientation. This single crystallinity was kept until some grains appeared at the top of the specimen after four depositions. The crystal orientation was approved by the EBSD map given in Figure 5b, where the substrate and the deposited part have the same color. This is characteristic of a single crystal, and no grain boundary could be detected at the interface between the substrate and deposition. The misoriented stray grains were observed clearly close to the top, suggesting that the single crystallinity was broken there. For substrate B, the microstructure and its EBSD map are given in Figure 5c,d. Since the laser metal deposition was conducted on the (101) surface along the [10$\bar{1}$] direction, the primary truck could not be seen. A large magnification of the dendrite is shown in the Figure 5e and the microstructure in the longitudinal section is given in Figure 5f, revealing that the dendrites grew well in the same direction as the orientation in the substrate. No stray crystals could be found in the epitaxial growth region, indicating that the dendrite maintained nice single crystallinity similar to that in the substrate, and it was validated as the EBSD map presented in Figure 5d. For substrate B, the single crystallinity could be extended to the top of the deposition, which was much better than that of substrate A.

### 3.2. z-Step of 300 μm in LMD

**Figure 6:**
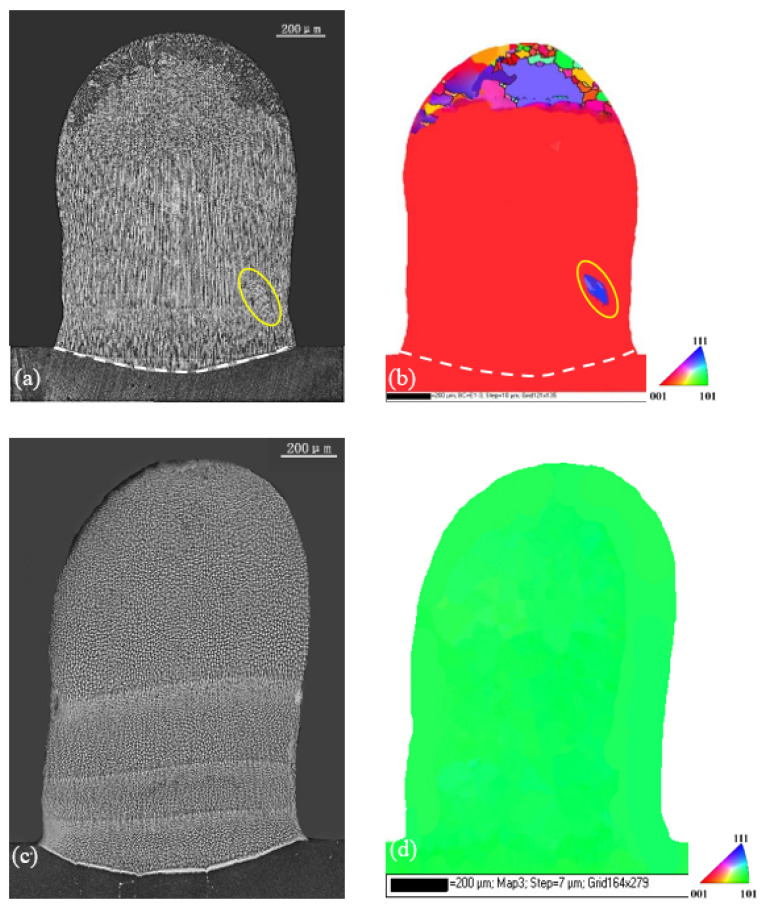
Microstructures and corresponding EBSD maps of substrate A (**a**,**b**) and substrate B (**c**,**d**) when z-step was 300 μm for three LMD layers.

If the z-step varied from 100 to 300 μm, the optical micrographs and the corresponding EBSD maps of the specimens in transverse section for the two substrates are given in Figure 6, where four layers are deposited with the final dimensions of 1.5 mm height and 0.8 mm width. For substrate A, it can be seen that a stray grain formed in the center part of the weld pool, as indicated by the yellow circle in Figure 6a,b. Due to the larger layer step, this stray grain was not remelted in the later treatment and grew continuously from the first trace to the second one. In the upper part of the sample, more stray grains appeared due to CET near the surface. Clearly, for this substrate, the increase in the layer step induced the stray grain formation. However, for the substrate B, although the layer step increased to 300 μm, the whole deposited part still maintained ideal single crystallinity, as shown in Figure 6c,d. 

### 3.3. z-Step of 300 μm in LR + LMD

**Figure 7:**
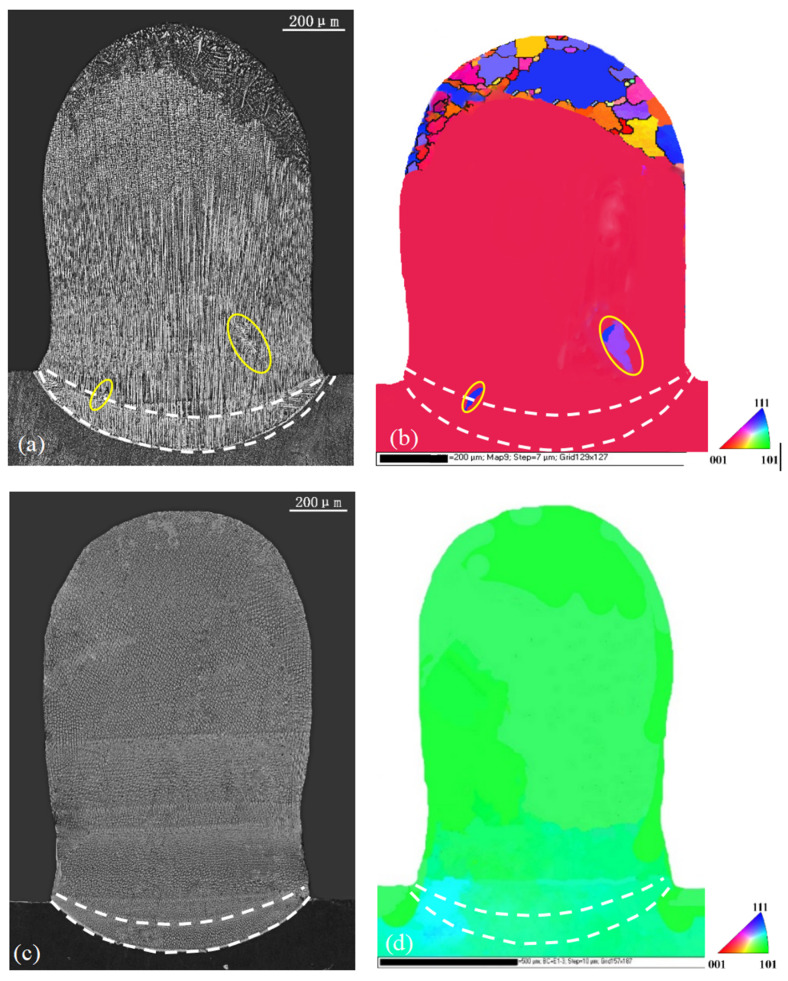
Microstructures and corresponding EBSD maps of substrate A (**a**,**b**) and substrate B (**c**,**d**) when z-step was 300 μm for LR plus LMD layers. The dash–dot line in (**a**) marks the [001]/[100] boundary in LR trace.

The optical microstructures and the corresponding EBSD maps of the specimens in the transverse section for the two substrates in the second set of experiments are given in Figure 7. Under the same processing conditions, the LR process produced a slightly larger remelted zone with a width of 1000 μm and a depth of 300 μm. In the following LMD process, because part of the heat input was used to melt the powder, the width of the remelted zone decreased to about 800 μm and the depth of the substrate decreased to 100 μm. The difference in the orientations of the two substrates had no effect on the size of the remelted zone. 

For substrate A, the height of [001] dendrite zone in the center LR trace of the transverse section was about 100 μm (Figure 7a), and the bottom trace of the first LMD layer was higher than the [001]/[100] boundary of the LR trace. This shows that the [001]/[100] boundary formed in LR was not remelted by the subsequent deposition, as we expected. An apparent stray gain, marked by yellow circles as SGs in Figure 7a,b, developed from the [001]/[100] boundary during the LR process. It extended to the first LMD layer due to the epitaxial growth in the LMD process which was followed. This indicates that the boundary was vulnerable to stray grain, which, if not avoided, would be harmful to the following deposition. The other two big strain grains marked by white arrows also formed in the first LMD deposit and extended to the second and the third layers. To prevent this, we needed to use a small z-step or higher laser power to increase the heat input to remelt the [001]/[100] boundary and the stray grain there. The increased laser power was similar to the situation of decreasing z-layer step in a continuous multi-layer LMD deposition, but it may have changed the single-crystal nature. Thus, the more reliable method was to keep the same power but decrease the z-step. In this case, it led to lower efficiency in the repair process.

Under the same processing conditions, for substrate B, the [001] dendrite zone extended to the top of the original substrate surface during LR, and the bottom of the remelted trace in the first layer of LMD process was still in the [001] dendrite zone in the LR melt pool, as shown in Figure 7c,d. Since the [001]/[100] boundary did not exist, there was no stray grain formation in the following LMD process. In addition, as we stated before, for this kind of substrate, stray grains have lowest possibility of forming. That is to say, there are two advantages of using substrate B. One is that it decreases the formation ability of stray grain, and the other is that even if stray grains appear, they will be located at higher LMD positions near the top part of the specimen. This will be verified by our numerical results in the next section. In such a case, a large z-step can be selected to increase the efficiency.

## 4. Discussion

It can be seen that, to obtain a good SX nature, the laser metal deposition on substrate B with the optimized orientation can require a relative larger layer step than that on substrate A with the normal orientation. The larger layer step is important in LMD since it can improve the efficiency to a certain degree. The most key factor in increasing the layer step is to remelt the stray grain produced in the previous laser scanning in the following laser treatment; thus, it would indicate that the position of the stray grain formation can be changed simply by varying the substrate orientation from a (001) to a (101) surface. The underlying mechanism of this will now be analyzed. For a clear statement, we will first calculate the temperature field to examine the layer profile and growth velocity distributions in the LMD part. Then, the stray grain formation ability will be determined as a function of position in the sample for the different substrates, and finally, we will show why a larger layer step can be taken in substrate B.

### 4.1. The Layer Profile and Dendrite Region Distribution in Repair Traces

The layer profile in the deposited parts decides the distributions of the thermal gradient and growth velocity, and thus has a significant effect on the stray grain formation ability in repair traces. In order to obtain deep insight into the susceptibility to stray grain, we determined the layer profile according to the calculated temperature field in the deposited part, which could be compared to the experimental ones. In such a case, quantitative information of stray grain formation can be given. 

Under the current experimental processing conditions, i.e., a laser power of 250 W and a laser scanning velocity of 5 mm/s, the convection in the laser trace could be suppressed effectively, which was validated in the previous laser metal deposition process. In this way, we performed the calculations without considering the convection effect. 

**Figure 8:**
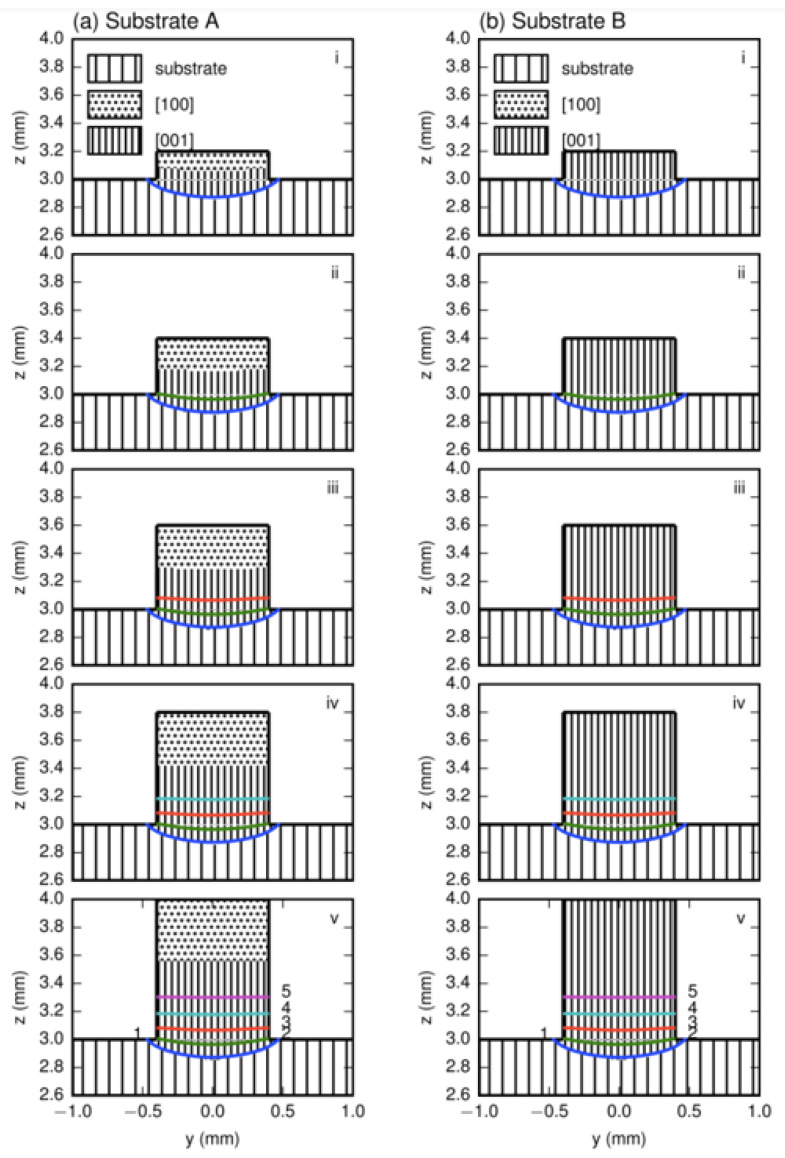
The preferred dendrite regions in (**a**) substrate A and (**b**) substrate B in the transverse section for LMD with 100 μm z-step. i to v in the figure denote the dendrite region distribution after the ith deposition.

**Figure 9:**
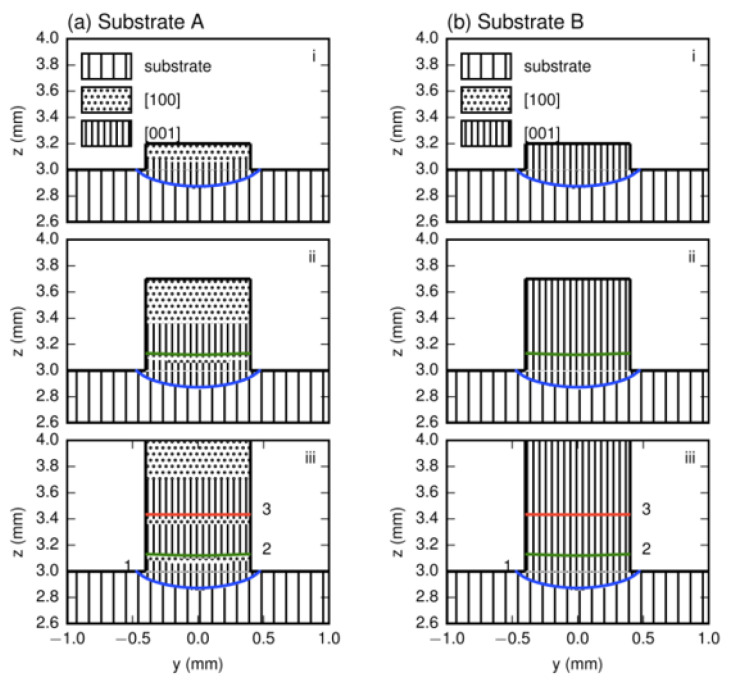
The preferred dendrite regions in (**a**) substrate A and (**b**) substrate B in the transverse section for LMD with 300 μm z-step. i to iii in the figure denote the dendrite region distribution after the ith deposition.

The temperature fields were calculated by using the parameters in refs. [7,8] and the results of the layer profiles in the transverse section for LMD with a 100 μm z-step and those for LMD with a 300 μm z-step for substrates A and B, which are presented in Figure 8 and Figure 9, respectively.

Since the thermal field did not vary with the substrate orientation, the layer profiles for certain z-steps were same for both substrates. For the two z-steps, the bottom trace for the first layer in the substrate showed a shallow curved shape, whereas others in the deposited part were nearly flat because the heat was mainly transferred to the substrate. The shapes of these lines corresponded well to traces during laser metal deposition processes, as shown in Figure 5, Figure 6 and Figure 7. 

Based on the temperature field, the dendrite regions with the preferred growth directions in the deposited layers could be determined. Due to the lack of the strong thermal dissipation in the two sides, [010] and [0$\bar{1}0$] regions did not exist, and only [001] and [100] regions formed. The [001] region appeared definitively in the lower part of each deposited trace, while the [100] region could form or not depending on whether the height of each deposited layer was higher than the [001]/[100] boundary or not.

For the z-step of 100 μm in substrate A, Figure 8a, in the first layer deposition, the [001] region forms in the lower part while the [100] region appear in the upper part. The [001]/[100] boundary is clear. After the second layer’s deposition, due to the small z-step, the whole [100] and partial [001] regions of the first layer are remelted and the new [001]/[100] boundary moves upward. When five-layer depositions are carried out successively, no [100] region exists within the first four layer traces in the lower main cladding part. This agrees well with the experimental result given in Figure 5a. When the z-step increases to 300 μm (Figure 9a), the bottom trace of the second layer cannot penetrate into the [001] region in the first layer; thus, the [001]/[100] boundary of the first layer is not eliminated. The situation is repeated after the third deposition proceeds. As for substrate B, for each deposition layer under the different z-steps, the [001] dendrite region grows and extends to the sample’s top; therefore, only the [001] region produces without any SGs no matter what z-steps are selected, as Figure 8b and Figure 9b show. 

The average heights of the [001]/[100] boundary and each layer, *h*_b_ and *h*_l_, can be determined from the calculated layer profile for substrate A. The z-steps are 100 and 300 μm, *h*_b_s are 230 and 250 μm, and *h*_l_ are 105 and 270 μm; see Figure 8a and Figure 9a. The average experimental layer heights for the two z-steps are determined from the four pervious layers to be 80 and 260 μm, values which agree well with the calculated data. 

Because the distributions of the preferred growth regions are different in the two substrates, the susceptibility to stray grain in the two cases is also varied, which will be determined next.

### 4.2. The Stray Grain Formation Ability in the Repair Structure

**Figure 10:**
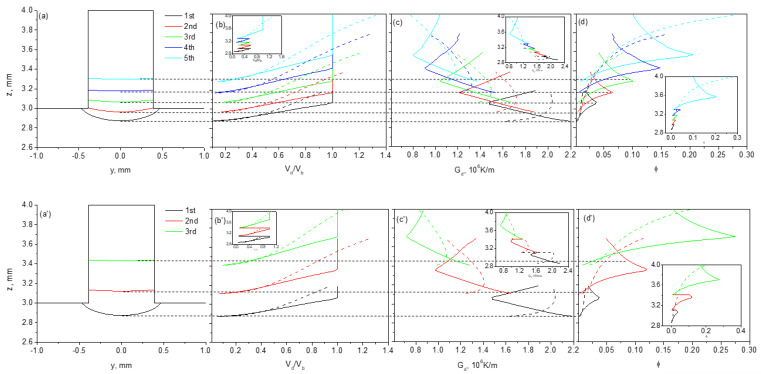
The calculated results of layer profiles, normalized growth velocity *V*_d_/*V*, the thermal gradient *G*_d_, and the average volume fraction of stray grain *ϕ* along the dendrite-preferred growth directions in the deposited part for multi-layer low z-step LMD (**a**–**d**) and for high z-step LMD (**a′**–**d′**).

Based on the calculated temperature fields, the normalized growth velocity *V*_d_/*V*, and the thermal gradient *G*_d_, the average volume fraction of stray grain *ϕ* along the dendrite-preferred growth directions in the deposited part can be determined. The calculated results are given as functions of center position in the transverse section of the laser metal deposition traces for 100 and 300 μm layer steps in Figure 10, in which solid lines denote substrate A and dashed lines denote substrate B.

**Figure 11:**
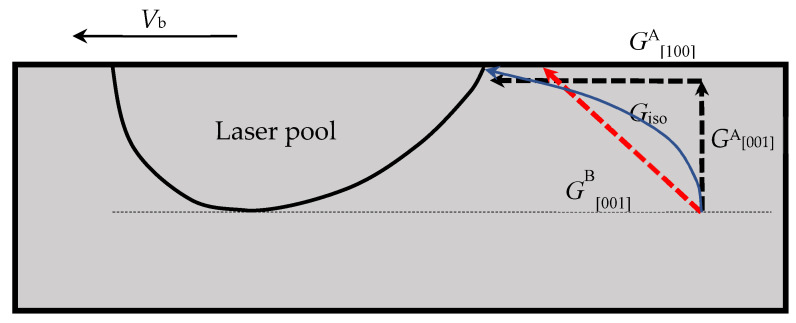
Schematic illustrations of thermal gradient directions along the isotherm and the preferred crystallographic orientations.

For the z-step of 100 μm in substrate A, with the increase in the z position from the bottom trace in each deposition, the normalized growth velocity of the dendrite increases firstly and is then kept at 1 from the [001]/[100] boundary, at which the [001] dendrite transitions to the [100] dendrite and the growth velocity along the [100] direction is the same as the laser scanning velocity. Correspondingly, *G*_d_ decreases initially and then increases with the height, and a turn appears at the [001]/[100] boundary. Since *ϕ* is characterized by $G_{d}^{n}/V_{d}$, it increases then decreases along the z axis in the layer if the next deposition is not performed, as shown in Figure 10d. *ϕ* has a maximum value at the [001]/[100] boundary where the stray grains have a higher probability of forming. It is obvious from the figure that this is because there is a minimum temperature gradient and a maximum dendrite growth velocity. With the successive depositions in this case, because of the small z-step, the [001]/[100] boundary of the previous layer is remelted by the next layer, except that of the fifth one. *V*_d_/*V*, *G*_d_, and *ϕ* cannot reach their maximum or minimum values in the previous layer then return to the values of the next one, as the insets are given. For the z-step of 100 μm in substrate B, however, the situations are different from those of substrate A, as the dashed lines given in Figure 10a show. In this case, because only the dendrite with the preferred growth direction of [001] exists, the growth velocity of the dendrite increases monotonously. For the thermal gradient along the preferred growth direction, it increases firstly and then decreases, which is quite different from the case in substrate A. This difference is induced by the 45° deviation between the two preferred [001] directions in substrate A and substrate B, and is illustrated in in Figure 11. The isothermal gradient *G*_iso_ is perpendicular to the isothermal lines during laser treatment, and it changes direction and decreases continuously. In substrate A, the direction of *G*_iso_ deviates from that of *G*^A^ [001] from the layer’s bottom in the [001] region, and then it approaches that of GA [100] from the [001]/[100] boundary surface in the [100] region. This leads to a “decrease-increase” tendency for *G*_d_^A^. In substrate B, however, since the direction of GB [001] for [001] dendrites is direct, as Figure 11 presents, first approaching and then deviating from that of *G*_iso_, it increases initially and then decreases. Because of these different characteristics of *G*_d_ in the two substrates, *ϕ* shows an “increase-decrease” tendency in substrate A, but increases monotonously in substrate B with z. However *ϕ* behaves in the two substrates, the most striking feature is that in each layer, it is much smaller in substrate B than in substrate A. 

For the z-step of 300 μm in the two substrates, as Figure 10b shows, the variation tendencies of *V*_d_/*V*, *G*_d_ and *ϕ* are similar to those for the z-step of 100 μm. The difference is that, due to the large layer height, the [001]/[100] boundary in substrate A still exists in the three layers, and *ϕ* is much higher than that in substrate B. It decreases after it experiences the maximum value; see the two insets in Figure 10d,d’.

On the basis of these calculated results, we now turn to discussing why the laser cladding efficiency can be realized in substrate B. 

### 4.3. The Underlying Mechanism for Improving Efficiency

Due to the difference in the substrate orientation, it is clear that the formation ability of stray grains in substrate B is much weaker than in substrate A at the same position in the epitaxial laser metal deposition process. This indicates that substrate B is more reliable for the repair of single-crystal superalloys. 

Normally, a repair is performed in a (001) surface, as given in substrate A, because the SX blades are usually casted in the [001] direction. If a surface defect appears, it is usually cut in a plane with a surface parallel to the (100) surface. In this case, (001)/[100], the crystallographic surface and orientation shown for substrate A in the present work, is employed to conduct the LMD repair. During this process, since stray grains are inclined to form in the [001]/[100] boundary, the next cladding is usually expected to perform and remelt the [001]/[100] boundary formed in the previous one. This could be realized by selecting a proper layer step. 

For substrate A, the average height of the [001]/[100] boundary is about 230 μm. If we use a z-step of 100 um, the layer height measures about 80 μm, which is lower than the boundary; thus, the [001]/[100] boundary is diluted and the risk of stray grain is suppressed. In this case, nice single-crystal growth is realized, as shown in Figure 5a. If the z-step of 300 μm is taken, the average layer height is measured to be 280 μm, and it is higher than the position of the [001]/[100] boundary. Therefore, the boundary formed in the previous layer cannot be remelted by current laser cladding. If stray grains form near the boundary, they will survive and break down the single-crystal nature. This situation can be observed in Figure 6a and Figure 7a, where stray grains form and grow into the following cladding cycles.

For substrate B, no [001]/[100] boundary exists, and the repair is safe for the 100 μm z-step in LMD, as shown in Figure 5c. Moreover, due to the lower formation ability of stray grains, the single-crystal area can be extended near the sample surface. Even if we increase the z-step from 100 to 300 μm, the single-crystal integrity can be kept well, as shown in Figure 6c. For this kind of repair, one can cut the defect part along the (101) plane from the top using a turbine blade, although the blade has a [001] main axis. This is not a problem from an engineering point of view. After the repair, the final direction along the main axis of the blade is still [100].

From the above analysis, it can be seen that the z-step in substrate A is limited by the height of the [001]/[100] boundary, which is a function of the substrate orientation. As in substrate B, a large layer step can be selected. This orientation effect can be used to improve the LMD efficiency, and this is what we emphasize in this paper.

## 5. Conclusions

The fact that the susceptibility to stray grain can be adjusted by the substrate orientation under the fixed external parameters such as laser power and laser beam velocity in the laser remelting process is applied to the laser metal deposition process. 

It reveals that, for the purpose of optimizing the substrate from an initial state to 45° around the [010] axis, LMD performs on the (101) surface and the laser beam scans along the [10$\bar{1}$] direction, and the layer height can be increased. 

The underlying mechanism is that this substrate orientation changes the distribution of the [001] dendrite region with respect to the [100] dendrite region. The [001]/[100] boundary is thus lifted to a high position in the deposited layer; then, layer height can be increased. The function of the lifting of the [001]/[100] boundary in each layer not only decreases the formation ability of stray grain significantly, but also eliminates the appearance of the maximum susceptibility.

Our findings can offer deep insight into the effect of substrate orientation on susceptibility to stray grain in the LMD process, and this study could improve the efficiency of laser repair of single-crystal components. 

## Figures and Tables

**Table 1:** Nominal compositions of superalloy DD6 in wt. %.

Element	Cr	Co	Mo	W	Ta	Re	Nb	Ti	Al	Hf	Fe	Zr	S	Ni
DD6	4.3	9.0	2.0	8.0	7.5	2.0	0.5	-	5.6	0.1	-	-	-	Bal.

## Data Availability

The original contributions presented in the study are included in the article, further inquiries can be directed to the corresponding authors.
